# Limitations of Common Bacteriological Media

**Published:** 1920-01-24

**Authors:** 


### THE FUTURE OF PATHOLOGY.

### I. Limitations of Common Bacteriological Media.

It has lately been brought prominently to the notice of even the least interested in the principles of bacteriological technique that there are among the disease-producing organisms a very high percentage which are at present impossible or extremely difficult to cultivate artificially outside the human body. The effect of this is not only to leave a regrettable gap in our knowledge of the particular pathological condition, but, far more serious, to leave the profession seriously handicapped in its attempts at immunity production and specific cure. The specific drug has proved invaluable in infections such as syphilis and amœbic dysentery, where the *rôle* of the arsenobenzols and emetine respectively are sufficiently well known to receive repeated prominence in the lay descriptions of medical triumphs. Similarly, and much more rightly, should the specific anti-sera, as in the instance of diphtheria and tetanus, appeal to the public for their remarkable and direct disease-neutralising properties. Failure in cultivating does not so much affect the experimental finding of the specific drug, but very definitely does it influence the production of the specific anti-serum.

Also, we should for a moment recall that, with relatively few exceptions, the use of vaccines has been disappointing in the *treatment* of disease once established. Prophylactically they have been the greatest boon, and, if we include the use of an attenuated but unidentified virus as adopted with vaccination in smallpox and the Pasteur treatment of rabies, we must admit that much has been accomplished, even when the causal organism has not even been defined, far less artificially cultivated. On the other hand, one of the several limitations, and one, in fact, that has so long continued that by now bacteriologists themselves may be apt sometimes to forget its magnitude, is that in so many apparently microbic diseases we cannot, except on the rarest occasions, produce any phase of artificial multiplication of the causal germ.

### Some Recent Developments.

Admittedly we have touched, in these few words, on only one of a host of medical consequences which will come to mind, but this one is given in the hope that it may typify the result of a great weakness in the pathology of to-day and illustrate the value we may in future expect to arise from certain advances outlined below.

In the first place, it is a matter of common knowledge that, particularly at the sites of pathological change in the human body, the reaction (acid or alkaline) is by no means necessarily the same as that normal to the healthy tissues. The divergence may be small but we are dealing with, in the single unit, extremely small causes and hence attention has recently been drawn to the reactions of artificial media. To a process of extreme refinement and standardisation, rather than any radical change, has effort been devoted, and the elements of the results at least should be in the possession of all who would claim any reliability for their laboratory findings.

### The Reaction of Media.

Repeatedly may it be noticed that although some bacteria will grow in media having a wide range of reaction, there are certain reactions at which an optimum growth occurs. On the other hand, there are certain organisms which require an extremely limited range, and to obtain any growth at all the reaction must be accurately defined within these limits. And lastly, this accurate adaptation may aid the growth of any particular germ at the expense of others.

This much can be proved every day in work with the known organisms, and perhaps it is not too much to hope that yet further refinement and with it clearer differentiation may come. To this end progress has already been made and the approved process of the Medical Research Committee outlined below has, to our own knowledge, already produced suggestive results.

To prove the gross inaccuracy of the methods formerly in vogue, let us note that, "Eight careful observers, using the same apparatus and the same medium, obtained results varying between 0.7 c.c. and 5.1 c.c. of a N/40 NaOH solution in titrating a 5 per cent. peptone solution"! If under carefully controlled conditions the differences were so great, it can be safely assumed that workers in different laboratories would encounter even wider discrepancies. The most obvious causes are doubtless the personal equation of the manipulator, his colour sense, and so forth, but the indefinite end-point is the root of the trouble.

### Hydrogen Ion Concentration.

The physical chemist will explain that the true reaction of any fluid is determined by the amount of hydrogen or hydroxyl (OH) ions which are present in it. These properly may be expressed as a concentration, and when we use this method to check the results of our old technique let us note, "A 'neutral' broth was prepared, using the titration method to determine its reaction. It was found to have a PH of 8.4. In other words, there is a difference between $1.0 \times 10^{-7}$ (=neutrality) and $0.04 \times 10^{-7}$, or a difference in hydrogen ion concentration of 1 to 25. It is evident that errors of this magnitude may readily give rise to difficulties—or mistakes—when the bacteriologist is dealing with organisms which will only grow well within somewhat narrow limits of reaction." Therefore we may conclude that this *true* reaction must be determined in place of the titration figure formerly in common use, provided that this can be done simply and expeditiously. In practice it can be, but we must refer the reader to the

**The Future of Pathology** –(*continued*).

Research Committee's own publications where the clearest details are provided. Suffice it here to say that by the use of a hydrogen ion electrode, accurate determinations of H ion concentration may be made, and these when made in the presence of a series of delicate colour indicators provide a series of permanent controls against which matchings of the medium may be made. In this way by an eminently simple process any desired point between PH 1.2 and PH 9.6 can be obtained with the greatest accuracy.

ACTION OF THE GROWING BACTERIA.

It is a fully recognised fact that many organisms produce *during* their growth an abrupt change in the reaction of the medium. The effect of this on the ideals of the above procedure and in the swamping and killing off of a delicate organism by a vigorous common saphrophyte growing alongside it is obvious. Nevertheless, even at this point of difficulty much assistance has been brought in by the recognition of certain well-known and simple compounds which can act as so-called "regulators" or "buffers" in the process of maintaining the reaction standard in spite of early bacterial by-products. These compounds have the property of taking up an excess of hydrogen ions and among their number we find many proteins, amino acids, and soluble salts such as phosphates, carbonates and borates. Again, space precludes the giving of any great detail, but we would call attention to the very marked advance that this type of accuracy denotes. True, it leads apparently to more complication in technique, more time spent in each laboratory investigation, but it has not only shown up many failings of the past, but also clearly indicated the path towards reform, and perhaps even a new era in bacteriology.

There is more in this work than the mere *reaction* of the medium.

FUTURE SYNTHETIC MEDIA.

IN what has been written above the best indication lies in the appreciation of "buffer" compounds and their effects, but let us for a moment recall what we remember of the past history of this science. Media galore, media of every sort and description, from rabbit's lung to a potato wedge or pea flour, have been used, and, for some particular purpose, with reasonable success. But how many of these choosings have been of an utterly empirical nature? How relatively few have been guided by more than a crude striving to imitate the natural condition, to imitate like with like! It is a principle which has served the profession well, but soon there is every hope that it will give place to the true synthetic medium. There the protein units, the amino acids, and the salts may be adjusted at will and with an eye to graded and exact requirements of the organism. Much of the work is already done and soon we hope to see the outcome a matter for wider publication. At first its use must inevitably be limited mainly to the research worker, but, like every other scientific development, each year should bring the synthetic medium into greater availability, and with it may come some degree of that urgently needed simplification in technique and germ differentiation, which is perhaps the most pressing requirement in the modern pathological laboratory.

For the moment, however, the practical worker must consider the published facts. There is no doubt that the latest advice on the standardising reaction of media should be followed in even the most humble laboratory unit. In practice the determination is, if anything, simpler than the time-honoured burette work, and as to its detailed adoption we may quote in summary :—

"Gelatin may be easily determined if the medium is liquified at 30°C. As a rule gelatin is distinctly acid in reaction, and its addition to broth causes a rise in the hydrogen ion concentration of the mixture.

"Good agar added to broth has but little effect on the latter. The reaction of the media may be estimated in the liquified media, but the temperature at which this is done makes the determination difficult. It is, therefore, better to use a clean agar which, preferably, has been washed, and take the reaction of the broth as that of the complete medium."

All bacteriologists have noted that media becomes more acid on sterilisation, and this difficulty is increased when a sugar is among the constituents. The simple methods by which it may be satisfactorily overcome are clearly explained in the Medical Research Committee's publication.

